# Maternal and child health handbook to improve continuum of maternal and child care in rural Bangladesh: Findings of a cluster randomized controlled trial

**DOI:** 10.1371/journal.pone.0266074

**Published:** 2022-04-06

**Authors:** Ruoyan Gai Tobe, Syed Emdadul Haque, Sanzida Mubassara, Rushdana Rahman, Kiyoko Ikegami, Rintaro Mori

**Affiliations:** 1 Department of Social Security Empirical Research, National Institute of Population and Social Security Research, Tokyo, Japan; 2 Department of Health Policy, National Center for Child Health and Development, Tokyo, Japan; 3 UChicago Research Bangladesh (URB), Dhaka, Bangladesh; 4 Department of Botany, Faculty of Biological Sciences, Jahangirnagar University, Dhaka, Bangladesh; 5 Department of Obstetrics & Gynecology, Dhaka Medical College Hospital, Dhaka, Bangladesh; 6 School of Tropical Medicine and Global Health, Nagasaki University NCGM Satellite, Tokyo, Japan; 7 Graduate School of Medicine, Kyoto University, Kyoto, Japan; Aga Khan University, PAKISTAN

## Abstract

This study aimed to evaluate the effectiveness of maternal and child health handbook (MCH) enhanced by mobile tools and to generate evidence informing the adoption of the program in Bangladesh. A cluster randomized controlled trial (RCT) has been implemented in Lohagora of Narail District and Dhamrai of Dhaka District. Unions of the study settings were randomly allocated in either one of three groups: (1) Intervention 1 using both mobile platform and MCH, (2) Intervention 2 using MCH alone, or (3) the Control. A total of 3,002 participants were recruited. The interventions were designed to promote two-way communications between pregnant women/their families and community health workers by an empowering approach. A total of 3,002 pregnant women were recruited. As the results, the interventions both significantly improved the utilization of CoC, although the overall proportion of CoC was relevantly low: 2.79% in the Control (95% CI: 1.37–3.54%), 6.16% in Intervention 2 (95% CI: 4.67–7.86%), and 7.89% in Intervention 1 (95% CI: 6.29–9.90%). Neonatal mortality rate with and without CoC was 5.43 per 1,000 (95% CI: 3.63–9.57 per 1,000) and 34.8 per 1,000 (95% CI: 24.3–45.4 per 1,000), respectively. Our study indicated the effectiveness of the interventions by leveraging MCH and a mobile platform to promote uptake of CoC throughout prepartum, intrapartum and postpartum/neonatal periods, potentially bringing long-lasting benefits to mothers and their offspring. The explicit approach is expected to guide policy makers to adopt MCH interventions in primary healthcare strengthening at the community level.

**Trial registration:** UMIN000025628 Registered June 13, 2016.

## Introduction

Although Bangladesh has achieved a significant progress on reduction of maternal and child mortality during the past decades, unmet targets on delivery and utilization of maternal and neonatal healthcare services have left the issue in an agenda of Sustainable Development Goals (SDGs). The new goals of the SDGs are to reduce the maternal mortality ratio (MMR) to less than 70 per 100,000 live births and neonatal mortality ratio (NMR) to 12 per 1,000 live births by 2030 [1–3]. So far, the effectiveness of interventions for saving the lives of mothers and babies have been proven [2, 4–7], but challenges remain in health-care seeking and practices across the full continuum of maternal and child care, including the utilization of antenatal care, birth with a skilled attendant or standard facilities, emergency obstetric care in case of complications or illness for women and newborn, essential neonatal care, and postnatal visits for women and babies in resource constrained settings [8–10].

The World Health Organization (WHO) [11, 12] recommended a home-based maternal record (HMR), an effective tool to actively link pregnant women and their families to community health workers and professional hospital staffs, raise knowledge and awareness on maternal and child health, identify complications in pregnancy and labor and common illness of mothers and babies, and consequently to improve delivery and utilization of maternal and child care services [13–20]. Among antenatal notes, immunization cards, child health books and the integrated document, the maternal and child health handbook (MCH) is the most comprehensive home-based book that encompasses all the records of the continuum of care for both mothers and children, including antenatal care, labor and delivery, postpartum care, newborn and child care, immunization and family planning. The integration of the different types of records is much more effective compared to the fragmented implementation, saving both financial and human resources for the intervention [21, 22]. Besides the records, the handbook also contains guiding information on seeking care for mothers and children conveyed through ample illustrations. The recently launched WHO guideline has recommended the use of home-based records to complement facility-based records [23]. So far, its effectiveness to improve health seeking behaviors, home care practices, male involvement and communication between health professionals and women / caregivers, and feasibility has been proven by empirical epidemiological studies in various developing settings [15–20]. However, there was insufficient evidence on the type, content and implementation of home-based records (HBR), which needs to be tailored to different sociocultural and epidemiological contexts [23].

In Bangladesh, a pilot MCH project showed strong positive impact on mother’s knowledge, practices, record keeping, service utilization and empowerment of women [24, 25]. After the approval by the Government of Bangladesh, a project-based utilization of HBRs has been widely implemented by NGOs; however, the current system of HBRs is fragmented, with various types provided by different organizations. Therefore, we implemented the first cluster randomized controlled trial (RCT) to examine the effectiveness of MCH enhanced by a mobile platform in two counties of rural Bangladesh (protocol available at: https://www.ncbi.nlm.nih.gov/pmc/articles/PMC5902947/) [26]. The existing version of the Bangladeshi MCH designed by Bhuiyan et al. were used in the intervention. The reason for enhancing it with a mobile platform was to boost communications between pregnant women, their family and community health workers, principal healthcare providers in the rural area, and to catalyze the potential advantages of the mobile platform in knowledge dissemination, guidance and promotion of healthcare utilization [27]. We hypothesized that the proposed interventions will benefit the continuum of care and lead to better maternal and neonatal outcomes. The study aimed to assess the effectiveness of the interventions on the improvement of the target outcomes, in order to inform updates of the MCH in the context of Bangladesh and policy making for the targets of SDGs related to maternal and neonatal health.

## Methods

### Study settings and participants

The community-based cluster RCT (trial registration: UMIN000025628) was conducted in two upazilas (administrative regions in Bangladesh), Dhamrai in Dhaka District, Dhaka Division and Lohagora in Narail District, Khulna Division from February 2017 to August 2018. The demographic characteristics of the study sites was summarized in Supplementary file (S1 File). The study period covered the duration from the start point at which the pregnant women were identified and recruited to the end point, when the participants came through the fourth week after giving birth, or terminated the pregnancy due to miscarriage, stillbirth and neonatal / maternal mortality. The cluster, namely the unions in each Upazila, rather than the individual, was subjected to the cluster randomized sampling. These unions in the targeted study sites have homogeneous socioeconomic characteristics such as income level of household, annual birth rate, accessibility to primary healthcare, literate rate, school attendance, and hygiene conditions in household. The sampling and randomization process was briefly described in the protocol and Supplementary file (S1 File). The selected unions were randomly allocated to either 1) the intervention that combined mobile phone communication with MCH, 2) the intervention using MCH alone or 3) the control, where the proposed intervention was voluntarily implemented after the study.

The target population was pregnant women aged 15 to 49 years living in the selected settings and expected to give birth between 1 August 2017 and 31 July 2018. The eligible criteria include: i. currently having a good health status, without any maternal complication; ii. living and planning to give a birth in the study settings during the period from February 01, 2017 to August 30, 2018 and iii. willing to participate to the proposed study with agreement to the informed consent. The pregnant women were basically identified from two routes: the registration list in the upazila health complex; and field activities of the community health workers (local NGO staffs), as they have close interactions with the rural residents.

The study also included healthcare providers at the community. We also included health professionals in each upazila as required. A total of 3,002 participants were finally recruited, including 998 for the intervention 1, 1,001 for the intervention 2 and 1,003 for the control. **Table 1** summarizes the participants in the study settings. Details of study design and sampling issues were described in our published protocol [26].

**Table 1:** Study settings and allocation.

Lohagora Upazila					Dhamrai Upazila				
Union No.	Control	Intervention 1	Internation 2	Total	Union No.	Control	Intervention 1	Internation 2	Total
1	0	0	113	113	13	0	103	0	103
2	109	0	0	109	14	0	0	86	86
3	0	0	126	126	15	0	74	0	74
4	0	0	79	79	16	87	0	0	87
5	0	0	190	190	17	71	0	0	71
6	0	222	0	222	18	0	0	107	107
7	141	0	0	141	19	0	0	81	81
8	78	0	0	78	20	92	0	0	92
9	96	0	0	96	21	0	88	0	88
10	0	126	0	126	22	0	0	109	109
11	76	0	0	76	23	0	156	0	156
12	0	144	0	144	24	76	0	0	76
					25	84	0	0	84
					26	0	0	110	110
					27	0	85	0	85
					28	93	0	0	93
**Total**	**500**	**492**	**508**	**1,500**	**Total**	**503**	**506**	**493**	**1,502**

### The interventions

The two interventions were designed to promote two-way communications between pregnant women/their families and CHWs by an empowering approach. The intervention 1 applied mobile platform, including text and / or audio messages and phone calls if necessary, which were combined with MCH, while the intervention 2 utilized MCH alone. Contents of MCH encompassed the general profile of pregnant mother, menstrual history and history of previous pregnancy (if any), records of health education and consulting, records of conditions/health status, healthcare utilization and clinical results during pregnancy, delivery and postnatal/neonatal period, as well as information on common complications and signs of danger, on health seeking for mothers and babies, and on daily care and nutrition. In the two interventions, MCH was distributed to each participant at the point of recruitment. Every two months the enrolled pregnant women and their families and CHWs were organized for a community meeting, where health education, consulting/advice and anthropometric measurements were provided to accompany the discussions on seeking health services for mothers and babies and the application of MCH. Additionally in Intervention 1, besides MCH and community meetings, user-friendly mobile messages were developed and sent according to the gestational age (GA), including reminders of antenatal and postnatal care visits and facility-based delivery, list of locations of skilled birth attendants and hospitals, GA-specific health issues, daily care and nutrition during pregnancy, intake of iron tablet and folic acid, support from husband and families during pregnancy and lactating period, signs of danger, signs of labor, and postnatal/neonatal care. Audio messages and phone call were also used for follow-up and consulting/advice, as necessary. For those participants in households without mobile phones, the community staffs made regular visits to their home according to their GA to provide equivalent information.

The primary study target was the pregnant women, and their families (husbands, mother-in-law and / or mothers) were also invited to participate to the interventions, as they were substantially involved in daily home-based care during and after pregnancy, as well as seeking and utilization of healthcare services during pregnancy, childbirth and neonatal period in the study settings. By leveraging the community network, pregnant women and their families were organized for health education / health promotion activities, and a linkage with the community health workers were established to provide advice and referral when necessary. Through the network built, the interventions also closely involved communications among health care providers at different levels: for example, those healthcare staffs at the community were able to provide supports and advice and refer those with maternal complications to health professionals at the upper-level facilities (Upazila and District), and health professionals at the referral level were able to provide necessary guidance and training as well. The study procedure was briefly described in the protocol and Supplementary file (S1 File).

### The expected outcomes

The expected outcomes were neonatal death, fetal death (stillbirth/miscarriage), preterm birth, low birthweight, maternal pregnancy complications and referral, antenatal care visits for at least one time (ANC1), antenatal care visits for at least four times (ANC4), antenatal care visits for at least six times (ANC6), facility-based delivery (FBD), mode of delivery, utilization of postnatal/neonatal care (PNC), and the experience of health education. The definition of neonatal deaths followed the standard employed by WHO, that is, death within the first 28 days of life. By referring Bangladesh Demographic and Health Survey (BDHS) 2014, neonatal deaths and fetal deaths were determined from the complete birth history from mothers and recorded by our trained staffs [28]. The continuum of care (COC) for mothers and babies in the study referred to healthcare services during pregnancy, at birth and after birth, and the variable was then created by combining ANC4, FBD and PNC.

### Data analysis

For data analysis, univariate analysis was first performed to explore the characteristics of variables. In the comparison of each variable, the equality of covariates of the three groups at baseline was examined by a stratification. To examine the effect of the interventions on the targeted outcomes, we carried out multivariate logistic regressions. We considered potential correlations in the participants from the same unions. Then, generalized estimation equations (GEE) with an independent correlation structure were performed. The covariates were adjusted by using ratio residuals for each cluster obtained from the logistic regression models. For the fixed effect and the mixed effect of the cluster, namely the unions, we also performed generalized linear mixed models (GLMM) for comparison, in which the union ID was subject to the randomization. Risk ratios (RR) and their 95% confidence interval (CI) were calculated for the targeted outcomes in the intervention groups compared to the control group, after controlling demographic and socioeconomic factors of the pregnant women, such as age, education, household income, and those related to healthcare accessibility. Data analysis was performed using Stata 15.0.

### Ethical consideration

The study was approved by the ethical committee of Bangladesh Medical Research Council (BMRC), Bangladesh. After stipulating the objectives, procedures, risks, benefits, confidentiality and voluntary both verbally and literally, the pregnant women who were willing to participate were asked for a signature in the informed consent form. For those unable to read and write, their substitutes (basically their families, relatives or neighbors) were asked to sign on the form on behalf. Additionally, for those aged below 18 years, their guardians were asked to accompany with, and then sign on behalf of them for the informed content. These consent procedures as described were approved by the ethical committee.

## Results

### Demographic and maternal characteristics of the participants

**Fig 1:**
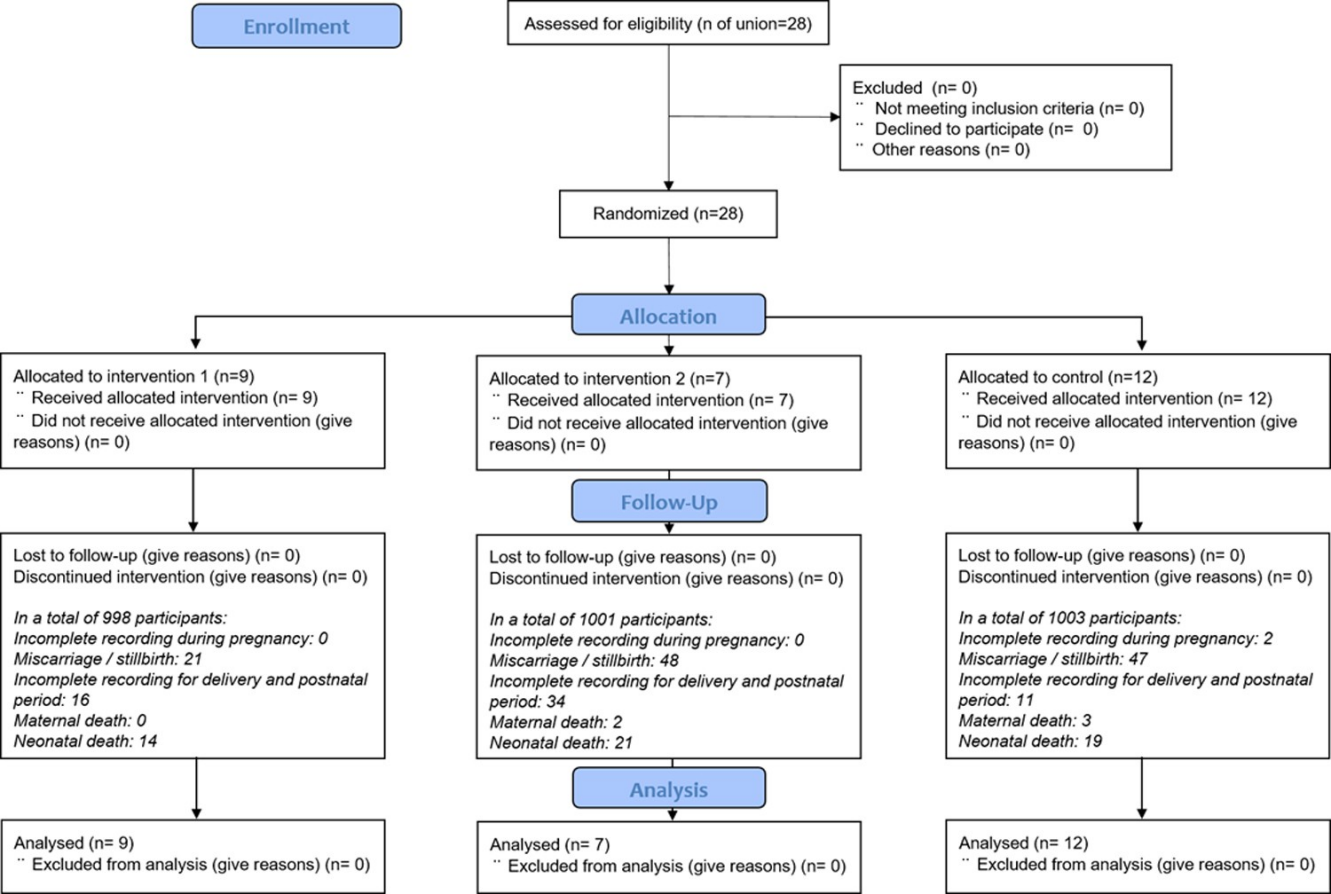
Participant flow diagram.

**Fig 1** summarized participant flow. The details of demographic and maternal characteristics of the participants were all listed in Supplementary file (S1 File). The average age of the participants was 23.53 years (SD: 4.67 years). The average gestation age at birth and birth weight was 37.4 weeks (SD: 1.8 weeks) and 2,841.24 g (SD: 439.64 g), respectively. The proportion of those live birth who undertook cesarean section was as high as 50.81%. Socio-demographical characteristics did not significantly differ between the intervention groups and the study settings at baseline.

### Mortality and morbidities

**Tables 2** and **3** generated results from univariate and multivariate analyses to examine effects of the interventions on the expected outcomes. Among the overall participants, there were 5 maternal deaths, 116 fetal deaths (miscarriage/stillbirth) and 54 neonatal deaths reported in the study settings. Neonatal mortality rate (NMR) was 27.8 per 1,000 (95% CI: 19.7–36.0 per 1,000), 28.2 per 1,000 (95% CI: 10.5–45.8 per 1,000), and 34.8 per 1,000 (95% CI: 17.6–52.1 per 1,000) in Intervention 1, Intervention 2 and control group, respectively, while no significant difference in the three groups was identified. The factors independently affecting neonatal survival included referral of complications during pregnancy and delivery, multiple birth, congenital malformation, maternal status and COC. NMR among those having received COC and the counterparts was 5.43 per 1,000 (95% CI: 3.63–9.57 per 1,000) and 34.8 per 1,000 (95% CI: 24.3–45.4 per 1,000), respectively.

**Table 2:** Univariate analysis for effects of the interventions on the expected outcomes.

		Odds Ratio	95% CI
**Antenatal care > = 1**	MCH+mobile vs. control	*1*.*199*	*1*.*142–1*.*259*
	MCH only vs. control	*1*.*116*	*1*.*060–1*.*176*
	interventions vs. control	*1*.*158*	*1*.*106–1*.*212*
**Antenatal care > = 4**	MCH+mobile vs. control	*2*.*344*	*1*.*733–3*.*170*
	MCH only vs. control	*2*.*233*	*1*.*648–3*.*027*
	intervention vs. control	*2*.*289*	*1*.*728–3*.*030*
**Antenatal care > = 6**	MCH+mobile vs. control	*2*.*344*	*1*.*127–4*.*876*
	MCH only vs. control	*3*.*509*	*1*.*751–7*.*031*
	intervention vs. control	*2*.*927*	*1*.*505–5*.*692*
**Postnatal care> = 1**	MCH+mobile vs. control	*1*.*169*	*1*.*052–1*.*300*
	MCH only vs. control	*1*.*125*	*1*.*010–1*.*252*
	intervention vs. control	*1*.*147*	*1*.*044–1*.*260*
**Facility of delivery**	MCH+mobile vs. control	*1*.*144*	*1*.*064–1*.*230*
	MCH only vs. control	*1*.*137*	*1*.*057–1*.*223*
	intervention vs. control	*1*.*141*	*1*.*069–1*.*217*
**Referral of complication in pregnancy and childbirth**	MCH+mobile vs. control	1.025	0.999–1.051
	MCH only vs. control	1.014	0.987–1.042
	intervention vs. control	1.019	0.995–1.044
**Survival status of mother**	MCH+mobile vs. control	1.003	0.999–1.007
	MCH only vs. control	1.001	0.996–1.006
	intervention vs. control	1.002	0.998–1.006
**Survival status of the newborn**	MCH+mobile vs. control	1.006	0.994–1.018
	MCH only vs. control	0.997	0.984–1.011
	intervention vs. control	1.002	0.991–1.013
**Low birthweight**	MCH+mobile vs. control	0.879	0.699–1.107
	MCH only vs. control	0.869	0.690–1.095
	intervention vs. control	0.874	0.718–1.064
**Continuum of care**	MCH+mobile vs. control	*4*.*735*	*3*.*080–7*.*279*
	MCH only vs. control	*3*.*382*	*2*.*164–5*.*286*
	intervention vs. control	*4*.*069*	*2*.*683–6*.*171*

**Table 3:** Multivariate analysis (GEE) for effects of the interventions on the expected outcomes.

	RR	95% CI		*p*
**Antenatal care> = 1**				
**MCH+mobile**	1.848	1.617	2.111	***0*.*000***
**MCH only**	1.450	1.276	1.649	***0*.*000***
**Control**	ref.			
maternal age	0.954	0.894	1.018	0.157
maternal education	0.998	0.960	1.039	0.937
household income	1.000	1.000	1.000	***0*.*002***
primiparous pregnancy	1.053	0.915	1.211	0.473
distance to the nearest facility	0.990	0.952	1.029	0.607
knowledge on healthcare seeking	1.551	1.364	1.763	***0*.*000***
**Antenatal care> = 4**				
**MCH+mobile**	1.573	1.325	1.869	***0*.*000***
**MCH only**	1.537	1.293	1.827	***0*.*000***
**Control**	ref.			
maternal age	0.999	0.924	1.080	0.980
maternal education	1.073	1.023	1.125	***0*.*004***
household income	1.000	1.000	1.000	***0*.*021***
primiparous pregnancy	1.011	0.857	1.192	0.895
distance to the nearest facility	1.041	0.994	1.090	0.088
knowledge on healthcare seeking	0.992	0.884	1.112	0.887
**Antenatal care> = 6**				
**MCH+mobile**	1.806	1.324	2.465	***0*.*000***
**MCH only**	1.446	1.047	1.996	***0*.*025***
**Control**	ref.			
maternal age	1.103	0.972	1.252	0.130
maternal education	1.128	1.044	1.218	***0*.*002***
household income	1.000	1.000	1.000	***0*.*001***
primiparous pregnancy	0.924	0.703	1.214	0.571
distance to the nearest facility	1.095	1.014	1.183	***0*.*020***
knowledge on healthcare seeking	0.973	0.794	1.192	0.788
**Postnatal care > = 1**				
**MCH+mobile**	1.358	1.185	1.555	***0*.*000***
**MCH only**	1.280	1.117	1.465	***0*.*000***
**Control**	ref.			
maternal age	0.984	0.922	1.052	0.642
maternal education	1.040	0.999	1.084	0.058
household income	1.000	1.000	1.000	***0*.*000***
primiparous pregnancy	0.889	0.773	1.022	0.098
distance to the nearest facility	1.010	0.971	1.050	0.621
knowledge on newborn care	1.820	1.661	1.996	***0*.*000***
baby’s sex	0.981	0.881	1.092	0.728
singleton or multiple birth	0.818	0.423	1.579	0.549
malformation	0.790	0.388	1.610	0.516
low birthweight	0.827	0.702	0.973	***0*.*022***
perceived health status of baby	2.436	2.194	2.704	***0*.*000***
**Facility delivery**				
**MCH+mobile**	1.280	1.087	1.508	***0*.*003***
**MCH only**	0.946	0.798	1.122	0.524
**Control**	ref.			
maternal age	1.010	0.930	1.098	0.811
maternal education	1.151	1.094	1.212	***0*.*000***
household income	1.000	1.000	1.000	***0*.*017***
primiparous pregnancy	0.849	0.711	1.013	0.069
distance to the nearest facility	1.021	0.973	1.071	0.394
antenatal care > = 4 times	2.995	2.294	3.911	***0*.*000***
complications during delivery	0.060	0.048	0.074	***0*.*000***
singleton or multiple birth	0.955	0.409	2.229	0.915
preterm	1.652	1.419	1.923	***0*.*000***
knowledge on complications / danger signs	1.048	0.902	1.219	0.538
knowledge on delivery	1.289	1.101	1.510	***0*.*002***
**Referral for complications**				
**MCH+mobile**	1.821	1.106	2.999	***0*.*018***
**MCH only**	1.500	0.945	2.381	0.086
**Control**	ref.			
maternal age	0.967	0.768	1.218	0.776
maternal education	1.215	1.042	1.417	***0*.*013***
household income	1.000	1.000	1.000	0.883
primiparous pregnancy	1.017	0.609	1.697	0.950
distance to the nearest facility	0.980	0.854	1.125	0.776
antenatal care > = 4 times	0.592	0.382	0.917	***0*.*019***
knowledge on complications / danger signs	1.128	0.813	1.565	0.470
knowledge on healthcare seeking	1.011	0.749	1.364	0.945
singleton or multiple birth	3.071	1.303	7.238	***0*.*010***
preterm	1.252	0.713	2.198	0.434
**Maternal survival**				
**MCH+mobile**	1.000			
**MCH only**	1.438	0.698	2.964	0.325
**Control**	ref.			
maternal age	0.758	0.503	1.140	0.183
maternal education	0.840	0.652	1.082	0.178
household income	1.000	1.000	1.000	0.425
primiparous pregnancy	1.461	0.587	3.637	0.415
distance to the nearest facility	1.042	0.800	1.358	0.761
antenatal care > = 4 times	1.000			
complications during delivery	1.000			
singleton or multiple birth	1.000			
preterm	0.950	0.399	2.259	0.907
knowledge on complications / danger signs	0.871	0.523	1.451	0.597
knowledge on guiding delivery	0.771	0.465	1.278	0.313
**Neonate survival**				
**MCH+mobile**	1.139	0.725	1.789	0.573
**MCH only**	1.131	0.725	1.765	0.588
**Control**	ref.			
maternal age	1.415	1.123	1.783	***0*.*003***
maternal education	1.047	0.933	1.174	0.436
household income	1.000	1.000	1.000	0.409
primiparous pregnancy	0.753	0.442	1.284	0.297
knowledge on daily care	1.306	0.277	6.164	0.736
knowledge on healthcare seeking	0.920	0.455	1.862	0.817
knowledge on complications / danger signs	1.605	0.713	3.608	0.253
knowledge on delivery	1.173	0.552	2.494	0.679
knowledge on newborn care	1.254	0.659	2.385	0.491
referral of complication during delivery	0.483	0.234	0.996	***0*.*049***
singleton or multiple birth	0.274	0.092	0.811	***0*.*019***
malformation	9.023	3.551	22.927	***0*.*000***
baby’s sex	0.820	0.573	1.174	0.278
low birthweight	0.755	0.364	1.567	0.451
preterm	0.929	0.643	1.343	0.695
maternal survival	0.265	0.075	0.934	***0*.*039***
cesarean section	0.725	0.453	1.159	0.179
***continuum of care***	0.273	0.118	0.630	***0*.*002***
**Low birthweight**				
**MCH+mobile**	0.877	0.731	1.051	0.156
**MCH only**	0.857	0.698	1.051	0.138
**Control**	ref.			
maternal age	0.961	0.893	1.034	0.289
maternal education	0.961	0.916	1.009	0.108
household income	1.000	1.000	1.000	0.512
primiparous pregnancy or not	0.952	0.782	1.158	0.620
antenatal care > = 4 times	1.056	0.925	1.207	0.419
delivery at hospital	1.136	0.903	1.429	0.275
baby’s sex	1.040	0.908	1.191	0.572
singleton or multiple birth	0.478	0.282	0.808	***0*.*006***
preterm	1.111	0.991	1.245	0.071
complications during delivery	1.035	0.856	1.252	0.720
**Continuum of care**				
**MCH+mobile**	2.197	1.743	2.769	***0*.*000***
**MCH only**	1.701	1.340	2.159	***0*.*000***
**Control**	ref.			
maternal age	1.006	0.918	1.104	0.891
maternal education	1.067	1.008	1.130	***0*.*025***
household income	1.000	1.000	1.000	***0*.*018***
primiparous pregnancy or not	0.980	0.806	1.192	0.841
distance to the nearest facility	1.026	0.971	1.083	0.361
knowledge on healthcare seeking	0.991	0.873	1.124	0.886
preterm	1.133	1.069	1.201	***0*.*000***
complications during delivery	0.577	0.492	0.676	***0*.*000***

### Healthcare seeking during pregnancy, at birth and after birth

During pregnancy, participants in Intervention 1, Intervention 2 and control group went to 2.01 times (SD: 1.40 times), 1.97 times (SD: 1.49 times), and 1.48 times (SD: 1.29 times) of ANC on average, respectively. **Table 4** summarized the major outcomes as predicted. The indicator of COC in Intervention 1, Intervention 2 and control group was 11.88% (95% CI: 9.91% - 13.85%), 7.80% (95% CI: 6.16% - 9.43%), and 2.79% (95% CI: 1.56% - 4.01%), respectively. Compared to the control group, the proportions of ANC1, ANC4, ANC6, and PNC were higher in the two intervention groups, and FBD and referral for complications during pregnancy were better in Intervention 1.

**Table 4:** The expected outcomes as predicted by multivariate analysis (GEE).

	%	95% CI	
**Antenatal care> = 1**	77.38	75.86	78.90
**MCH+mobile**	84.79	82.57	87.01
**MCH only**	78.54	75.98	81.09
**Control**	66.56	63.31	69.82
**Antenatal care> = 4**	11.06	9.90	12.22
**MCH+mobile**	13.36	11.24	15.49
**MCH only**	12.86	10.77	14.96
**Control**	5.96	4.32	7.61
**Antenatal care> = 6**	2.46	1.89	3.03
**MCH+mobile**	3.83	2.62	5.03
**MCH only**	2.36	1.43	3.30
**Control**	0.98	0.32	1.65
**Postnatal care > = 1**	42.36	40.73	43.99
**MCH+mobile**	45.66	42.86	48.46
**MCH only**	43.82	41.07	46.57
**Control**	36.37	33.38	39.36
**Facility delivery**	61.23	59.99	62.47
**MCH+mobile**	64.50	62.34	66.65
**MCH only**	59.89	57.76	62.02
**Control**	58.90	56.75	61.05
**Referral for complications**	98.24	97.49	98.99
**MCH+mobile**	99.01	98.13	99.90
**MCH only**	98.44	97.31	99.57
**Control**	96.26	93.80	98.73
**Neonate survival**	97.23	98.04	96.42
**MCH+mobile**	96.96	98.83	95.10
**MCH only**	97.44	98.38	96.50
**Control**	97.23	98.80	95.65
**Low birthweight**	12.75	11.52	13.97
**MCH+mobile**	11.98	9.93	14.02
**MCH only**	11.52	9.46	13.59
**Control**	14.79	12.49	17.08
**Continuum of care**	8.03	7.04	9.03
**MCH+mobile**	11.88	9.91	13.85
**MCH only**	7.80	6.16	9.43
**Control**	2.79	1.56	4.01

### Experience of health education

In the two intervention groups, all participants reported that health education was provided during pregnancy, while in the control group, there 36.5% participants reported no relevant experience. Regarding the (potential) usefulness of MCH for knowledge dissemination for mothers and babies, 99.9% and 81.2% of participants in the intervention and the control group, respectively, had a positive attitude.

## Discussion

To our knowledge, this study is the first cluster randomized controlled trial to assess the effect of the MCH program enhanced by mobile platform This is also the first cluster randomized design for HBR to improve COC for mothers and babies in Bangladesh. Our findings indicated that the application of MCH improved uptake of multiple healthcare services, including antenatal care and postnatal/neonatal care, among rural pregnant women. The interventions increased ANC visits by using MCH and combining MCH and mobile platform, respectively. Although the overall proportion of at least four visits of ANC as recommended was relevantly low in the study settings, the figure in the two intervention groups, especially in the combined intervention, were better. A similar tendency was also observed in PNC. The combined intervention further improved facility-based delivery and utilization of healthcare facility for complications during pregnancy and delivery. The multilevel GEE models identified statistical significance of these intervention effects, after adjusting potential confounders.

Compared to the monitoring data of UNICEF which targeted the overall population during the study period in Bangladesh [29], our study which targeted pregnant women living in rural areas identified higher NMR of 29.7 per 1,000 (95% CI: 21.6–37.8 per 1,000). Although the estimated figure was lower in the two intervention groups, no statistical significance on the immediate efforts to reduce mortality and morbidities was identified. A possible reason for this could be the calculation of the study sample size was based on an NMR of 24.4 per 1,000 (derived from the final MDG report), while the indicator has been substantially reduced since then. We also acknowledged that unlike obstetric care practices, MCH does not have an immediate life-saving effect and that the universal access to good-quality obstetric and neonatal healthcare plays a key role in reducing NMR based on the success observed in Bangladesh and other developing settings [30, 31]. On the other hand, consistent with the findings of a systematic review, [32] our analysis confirmed that a crucial determinant in reducing NMR was COC; both interventions showed a significant improvement. This suggests that MCH has a potential to improve neonatal survival through the promotion of utilization of COC for mothers and the newborn.

In our study, MCH brought upon several benefits, such as health education, promotion of daily care awareness and practices, involvement of husband and family members and boosting communication between pregnant women and healthcare providers, especially CHWs, leading to better healthcare utilization during pregnancy, at birth and after birth. This was compatible to previous studies on MCH [16–20]. The interventions involved primary healthcare at the community as an inevitable aspect. In the intervention settings, and the local residents, including pregnant women and their families, were organized and networked, and community meetings aiming to strengthen participatory learning and action on preventive and care-seeking behaviors were also implemented regularly. Similar empowerment practices have proven to be effective in improving key behaviors and neonatal survival outcomes, although its mechanism may depend on local practices, capabilities and the responsiveness of health services [33]. In our study, during this empowerment process, MCH or MCH combined with the mobile platform were the key instruments. CHWs were mobilized to reinforce the linkage, deliver knowledge and primary care, organize the community meeting and bridge pregnant women and healthcare facilities, in order to accomplish the proposed interventions. To this end, the results suggested that MCH can be a useful tool to strengthen primary healthcare delivery in rural Bangladesh. The interventions largely filled the gap of health education during pregnancy and routine primary healthcare at the community level, and the (potential) usefulness of these interventions were definitely recognized among most participants.

Compared to MCH alone, the combined intervention achieved better utilization of COC, especially in terms of facility-based delivery and care seeking for complications during pregnancy and delivery. What works for this intervention were likely to be effective contacts and more frequent interactions between pregnant women and CHWs, such as sharing information and advising daily home-based care, together with seeking relevant healthcare based on individual needs and requirement. Text and voice messages complemented MCH in knowledge dissemination and deepening the understanding of the key contents of MCH. The high mobile coverage and the low costs in the study settings facilitated the intervention. The results added relevant evidence on the effectiveness of mHealth on improvement of maternal and neonatal outcomes and related care seeking by the high-quality study design, which were of lack in low- and middle-income countries [34], and suggested the value to apply these effective tools in primary healthcare at the community level.

Our study revealed the latest status of universal health coverage for mothers and neonates in rural Bangladesh. The uptake of ANC4 among rural pregnant women living in the study settings was considerably lower than that of the overall population identified by BDHS 2014 [35, 36], but was comparable to that of community-based studies conducted in a rural area [37, 38]. This can be explained by a substantial rural-urban gap in the uptake of maternal healthcare services [39]. Contrary to the stagnant progress in ANC uptake, our results suggested a fairly progressive uptake of PNC and FBD compared to previous surveys and estimates [40, 41]. The overall low uptake of these maternal and neonatal services suggested a big room for improvement through strengthening primary healthcare as the frontline of health system [42], particularly in rural areas.

The incidence of cesarean section (CS) identified in our study was much higher compared to that in BDHS 2014 [43], and largely exceeded the optimal rate ranging from 5% to 20% [44], while we did not identify the significant impact of undertaking CS on neonatal mortality reduction. Although it is a life-saving measure in obstetric care, a high level of CS indicates a substantial proportion of the practice without medical indication, leading to wasting of scarce healthcare resources and a high health and economic burden, especially in low- and middle-income countries [45–47]. The mechanism of the high-level CS tended to be complicated, mixing motivations of both the supply and demand sides, and the decision of the mothers and their family may largely be affected by doctors due to poorly informed healthcare needs [43, 48]. Our results suggested that this alarming phenomenon is emerging in not only urban areas, but also in rural areas recently, and an intervention by applying MCH and mobile platform had the potential to reduce the misuse. The emerging issues on CS in MCH for implementing health promotion/health education programs at community level are expected to be covered.

In interpreting these major findings, several issues should be carefully considered. The enrollment of the target pregnant women relied on self-report and local registration. Because of the variation in identifying pregnancy among the participants, gestational age at enrollment was diversified, causing differences in the participation duration. Moreover, our study was likely to be inevitably contaminated somehow, because the interventions and the outcomes cannot be masked, and there had been some previous NGO-driven health promotion campaigns and activities targeting the rural community in the study settings. However, there was no differences regarding these factors across the study settings and groups. Finally, because of the limited follow-up duration, our study did not observe the outcomes posterior to the neonatal period, potentially missing the overall effects of the target tools on maternal and child health.

## Conclusions

In conclusion, our study indicated the effectiveness of the interventions by leveraging MCH and a mobile platform to promote uptake of COC throughout prepartum, intrapartum and postpartum/neonatal periods, potentially bringing long-lasting benefits to mothers and their offspring. These tools coordinated the interactions of pregnant women, their families and CHWs and their active engagement in primary healthcare at the community level, potentially contributing to better health outcomes. It is worth including these tools in primary healthcare to achieve universal health coverage for mothers and babies in rural Bangladesh.

## Supporting information

S1 Checklist CONSORT 2010 checklist of information to include when reporting a randomised trial*. (DOC)

S1 File Supplementary information about the study. (DOCX)

S2 File (DOCX)
