# Supplementary material for: Maternal and child health handbook to improve continuum of maternal and child care in rural Bangladesh: Findings of a cluster randomized controlled trial
Source: PLoS One. 2022 Apr 6;17(4):e0266074. doi: 10.1371/journal.pone.0266074 (PMC8986009; doi:10.1371/journal.pone.0266074)
Supplement: S2 File — (DOCX) [file pone.0266074.s003.docx]

**Mobile-health tool to improve maternal and neonatal health care in Bangladesh: a cluster randomized controlled trial with economic evaluation**

**Background**

Every year, approximately 290,000 women die due to pregnancy and childbirth, and 99% of maternal mortality occurring in the developing world. Bangladesh is one of those developing countries with high maternal mortality, standing at 170 per 100,000 live births. Although it has become a lower-middle income country with economic growth and achieved various targets of the Millennium Development Goal (MDG), the target on reduction of maternal mortality and utilization of related obstetric and reproductive services provided by skilled health personnel remains unmet, and the progress in reduction of neonatal mortality lag behind that in the reduction of infant and under-five mortalities, with the proportion of neonatal mortality accounting for 39% of under-five mortality in 1991 increasing up to 60% in 2012.

Accessibility to perinatal health care at both community and referral level, including antenatal care visit, timely referral for the complications and childbirth attended by skilled health personnel is crucial to reduce maternal and neonatal deaths. In rural Bangladesh, where the home delivery by traditional birth attendants accounts for approximately 90% of all child birth, use of antenatal care as recommended by the World Health Organization (WHO), skilled birth attendant, and obstetric and neonatal cares for complications at the referral facility urgently need to be improved. However, a recent case study showed that mobile birth notification system support to seek help during labour for delivery by the trained health workers. On the other hand, “Aponjon” (meaning dear one in Bangla) is the Mobile Alliance for Maternal Action’s (*MAMA*) program in Bangladesh aimed at reducing maternal and child mortality using the power of mobile communication technology funded by USAID to support the scale up of a commercially viable mobile phone service that delivers weekly stage based messages to pregnant women, new mothers and their families. There might have some other program based activities but we need the scientific evidences through real research.

Mobile phone use is universal in Bangladesh. Even in the rural area, where the fixed phones are much less available, mobile phone network coverage and ownership are widespread, expected to serve as a potential platform to implement interventions for a continuum of care throughout pregnancy, delivery, postpartum and infancy highlighted by the WHO. Major effects of a mobile-health program are to link pregnant women with related healthcare services at different levels, to record their health status throughout pregnancy, delivery, postpartum and infancy, and to provide necessary knowledge, information and guidance to pregnant women and their families.

The Maternal and Child Health (MCH) handbook originated from Japan has been proven as an effective tool to facilitate client-provider communication and information-sharing, to record health status, to raise health awareness, to identify maternal and neonatal complications, and to encourage health-seeking behaviors. It is an innovative trial to incorporate the MCH handbook with the mobile phone platform providing the maternal and neonatal health intervention, as all previous study proven the effectiveness of the MCH handbook in developing countries were paper-based, not mobile phone platform-based. The objective of the proposed study is to assess the effects of such the trial in rural Bangladesh by a high quality study design, aiming to inform health policy making of stakeholders at different levels regarding maternal and child health in Sustainable Development Goals (SDGs).

**Methods**

**Study design and location**

This community-based randomized controlled trial will be conducted in two Upazilas (sub-districts) in Bangladesh. These two Upazilas are Araihazar (from Narayanganj District in Dhaka division) and Lohagora (from Narail District in Khulna division). Araihazar has 10 Unions and Lohagora has 12 Unions (the smallest administrative unit in Bangladesh). Each Union has 20-25 villages with an approximate population of about 25,000 and is served by a primary-level healthcare facility. In *Araihazar* area, there are 78,478 house-holds in 196.95sq.km where 381,754 people are living. In this area, the University of Chicago and Columbia University, USA jointly started public health researches since 1999 and estabilsied an international NGO (UChicago Research Bangladesh (URB)) for global health research. URB research team focuses several global health concern including maternal and child health in Bangladesh. In *Lohagora* Upazila, there are 44,963 house-holds in 289.0sq.km where 227,454 people are living. Thus, we will conduct this RCT in the mentioned two Upazilas in the two districts in Bangladesh. The basic indicators are the same in the proposed areas and URB will take the lead for implementation of this RCT and management of the field work in Bangladesh. The use of mobile phone in the study settings is expanding.

**Study population / Participants**

Study population is pregnant women aged 15-49 years old living and planning to give a birth in the study settings during the period from October 01, 2016 to June 30, 2018 and willing to participate to the proposed study with agreement to the informed consent. Participants also include community health workers (CHW), skilled birth attendants (SBA), staffs from community clinic and support committee for community clinic at the community / primary level and health professionals (obstetricians / neonatal doctors / nurses) at the referral level (upazilas, districts). Written informed consent will be acquired from all those participants for permission to collect data.

**Figure 1. Study population and intervention**


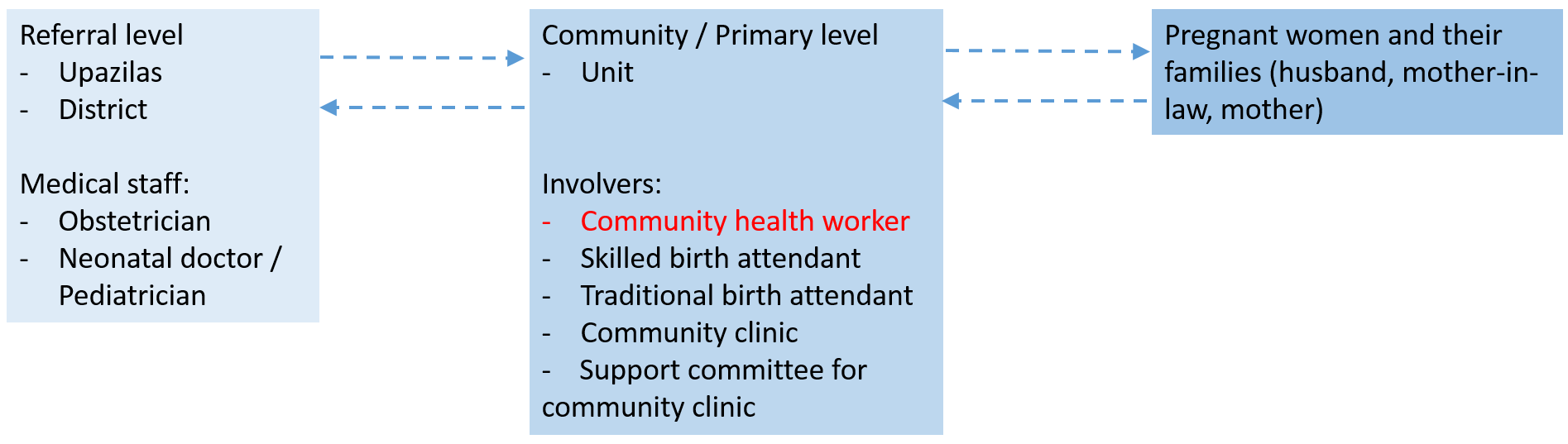


**Randomization and allocation**

We will include all units in the Upazilas except the sadar Upazila because of its better socio-economic status compared to others. There are 10 unions in Araihazar and 12 unions in Lohagora. Within each Upazila, 12 unions will be randomly divided into either intervention or control, 6 for each, respectively. There are 11 unions in the intervention group and 11 unions in the control group finally. We will start the intervention from one side of Upazila and other site will be control due to avoid contamination. All eligible pregnant women are subject to the recruitment. The expected sample size is 3,000, 1,000 for the intervention and 2,000 for control. Sample size is calculated using Epi Info 7.1.5 software.

**Study procedures**

Based on eligible criteria of the participants, our trained CHWs will visit each household to collect data as baseline information about the pregnancy of women from the village using a structured questionnaire. After baseline, we set-up the eligible participants details electronically. In the intervention group, recruited CHWs, TBAs / SBAs and field staffs will also receive one-week training from the maternal and child health experts and the health professionals working the referral level facility and get idea about their role in the community and necessary knowledge to provide basic cares, identify the complications and implement referral to the upper-level facilities. Then they will start to enroll pregnant women from the study sites. At the baseline survey, the demographic and socioeconomic information of each participant will be collected by a structured questionnaire after the consultation by the expert and a small-scale pilot in a village outside of the study settings. When the pregnant women enrolled, a code for the identification during the study period and the printed current version of Bangladeshi MCH handbook will be provided to them. In the intervention group, the platform of mobile phone short messaging and audio system will be used to provide related health information and advices as a continuum of care, to alert ANC and other healthcare services when necessary, and to communicate and seek helps between CHWs and pregnant women / their families (husbands, mother-in-law and mothers), and between CHWs and SBAs at the community level and health professionals at the referral level as well, as described below. Besides pregnant women, their families (husbands, mother-in-law and / or mothers) are also invited to participate to the intervention, as they are often the key person to affect health seeking, home-based cares and utilization of related healthcare services for pregnancy and childbirth in rural Bangladesh. CHWs play a central role to link pregnant women and their families and the referral-level facilities. Our trained field staffs will assist CHWs to take care of pregnant women in intervention group in the community as well. Through the mobile platform, every two month, enrolled pregnant women and their families, CHWs, village doctors and TBAs / SBAs will be organized to a community meeting, for face-to-face health education, consulting / advices and ANC during pregnancy. Data on health status in pregnancy, childbirth and postpartum, utilization of related healthcare services at community and facility levels, and maternal and neonatal outcomes will be collected by CHWs by using the mobile health platform, home visiting and facility visiting. Particularly for those not having mobile phone, home visiting by CHWs and communicating face-by-face is the only way to implement the intervention. The MCH handbook will be also recorded by CHWs. Because the MCH handbook is expected to record all related information, the end-of-point survey will not be required in the intervention group. In the control group, a routine care will be provided as usual and data will be collected by home visiting, facility visiting and the end-of-point survey.

**Intervention**

In the intervention settings, we plan to fully play the potential role of the platform of mobile phone short messaging and audio system and effectively link pregnant women and service providers involved in maternal and perinatal care at different levels: upazilas, unions, and villages. The intervention period is about 22 months (one year recruitment of participants + follow-up during pregnancy and the neonatal period) and expected to start from October 01, 2016 when we start to enroll the participants, to June 30, 2018 when all participants go through the neonatal period, four weeks after giving birth. The contents of the existing version of Bangladeshi MCH handbook will be incorporated into the mobile health program, with short message or audio message edited based on the gestational age and individual needs. The version of the MCH handbook is subject to review and the latest evidence-based practices recommended by WHO will be added appropriately. The major components of the intervention include:

- CHWs to pregnant women and their families: to provide information and health education for enhanced awareness on maternal and neonatal health; to provide advices as a continuum of care by linking SBAs and village doctors at the community / primary level and health professionals at the referral level; to alert and help to seeking ANC, SBAs and facility-based healthcare services when necessary to pregnant women and their families, particularly help to accessing to the emergency care; to identify complications or any condition needing referral;
- Pregnant women to CHW: to consult and seek advices in daily life;
- Pregnant women to village doctors and SBAs: to seek basic perinatal services;
- CHWs, village doctors and SBAs to health professionals at the upper-level facility: to consult and seek advices, to implement case referral;
- Health professionals to CHWs and SBAs: to provide training and inform the primary / community-level staffs, to effectively receive the referred cases

**Expected outcomes**

- Primary outcomes (collected by community health workers)
- neonatal mortality (deaths in the first 28 days per 1000 live-births)
- preterm birth, low birthweight
- maternal mortality
- maternal morbidities such as pregnancy complications or near miss as an alternative
- Secondary outcomes
- timely identification of maternal complications
- frequency of antenatal care visits
- accessibility to facility-based delivery
- referral for identified complications
- utilization of postpartum care
- status of initiating breastfeeding

**Quality control**

URB will take responsibility to maintain the quality of the implementation and data collection. The local program coordinator (s) will train the field staffs at the beginning of the project and provide enough education and refreshing training for the standard control as they are doing. Appropriate measures will be taken to prevent missing data, losing questionnaire and drop-out and to ensure valid sample size as mentioned above.

**Ethical consideration**

This study will be conducted after receiving approval from the ethical committee of Bangladesh Medical Research Council (BMRC), Bangladesh and National Center for Child Health and Development in Japan. The informed content will be implemented targeting all participants, and all paper-based and electronic-based personal information will be anonymous in data analysis.

**Timeline**

- Study preparation (document for the granting, study proposal, questionnaire, research agreement, ethical procedure, trial registration, transfer of budget): April - September 2016
- Field preparation (recruitment and training of local staffs, logistic preparation): September 2016 or right after the budget arrived
- Recruit of the first participant: October 2016 (expected)
- Recruit of the last participant: October 2017 (expected)
- Follow-up of the first participants for pregnancy and the neonatal period: October 2016 – June 2017 (expected)
- Follow-up of the last participant for pregnancy and the neonatal period: October 2017 – June 2018 (expected)
- Intervention and data collection by the local staffs: October 2016 – June 2018 (expected)
- Data cleaning and input: July - August 2018
- Data analysis, dissemination of the results, final report to JSPS and application of the next Grand-for-Aid: September 2018 – March 2019

**Research team**

1. **Principal Investigator(s):**

**Ruoyan Gai, MHSc, PhD**

Chief of Division of Policy Evaluation, Department of Health Policy

National Center for Child Health and Development of Japan

Okura 2-10-1, Setagaya-ku, Tokyo 1578535, Japan

Phone: +81.3.3416.0181; Email: [gai-r@ncchd.go.jp](mailto:gai-r@ncchd.go.jp)

**Syed Emdadul Haque, MSc, MHSc, PhD**

URB Coordinator of Research, Training, and New Program Development

UChicago Research Bangladesh (URB)

House # 4, Road # 2/B, Sector # 4, Uttra, Dhaka-1230, Bangladesh

Phone: +88 01712047987

Email: [emdad91@gmail.com](mailto:emdad91@gmail.com); [emdad@urb-bd.org](mailto:emdad@urb-bd.org)

**2. Co-Investigator(s):**

**Rintaro Mori, PhD**

Director of Department of Health Policy

National Center for Child Health and Development of Japan

Okura 2-10-1, Setagaya-ku, Tokyo 1578535, Japan

Phone: +81.3.3416.0181; Email: [rintaromori@gmail.com](mailto:rintaromori@gmail.com)

**Kiyoko Ikegami, PhD**

Professor, Graduate School of Social and Cultural Studies, Nihon University

Naktomominami 4-25, Tokorozawa City, Satatama Prefecture, 3590003, Japan

Phone: +81.90.4387.3351; Email: [ikegami.kiyoko@nihon-u.ac.jp](mailto:ikegami.kiyoko@nihon-u.ac.jp)

**Md. Tariqul Islam, MBSS, MPH**

Country Representative

UChicago Research Bangladesh (URB)

House # 4, Road # 2/B, Sector # 4, Uttra, Dhaka-1230, Bangladesh

Phone: +88 01713042651; E-mail: [tariqul@urb-bd.org](mailto:tariqul@urb-bd.org)

**Md. Mosiur Rahman, MSc, MHSc, PhD**

Associate Professor

Department of Population Science and Human Resource Development,

University of Rajshahi, Rajshahi-6205, Bangladesh

Phone: +8801910375448; Email: [swaponru_2000@yahoo.com](mailto:swaponru_2000@yahoo.com)

**Alauddin Ahmed, MBBS, MPH**

Chief Field Officer, UChicago Research Bangladesh

Tel: +88 01912191734; Email: [alauddin@urb-bd.org](mailto:alauddin@urb-bd.org)

**Data analysis**

To evaluate the effectiveness of E-health tool on maternal and neonatal health, we will compare the intervention and control groups’ through a cluster randomized trial. We will also compare between the study sites. Statistical analysis will be performed using statistical Software Stata 64-bit special edition, version 11.2 (StataCorp) and SPSS 16.0.

**Ethical consideration**

This study will be conducted after receiving approval from the ethical committee of Bangladesh Medical Research Council (BMRC), Bangladesh and National Center for Child Health and Development in Japan. The informed content will be implemented targeting all participants, and all paper-based and electronic-based personal information will be anonymous in data analysis.
